# Internal Consistency and Validity of a Short Spanish Version (10-Items) of the Center for Epidemiological Studies Depression Scale for Children and Adolescents (CES-DC)

**DOI:** 10.1155/2024/5409747

**Published:** 2024-09-25

**Authors:** María Eugenia Visier-Alfonso, Estela Jiménez-López, Eva Rodríguez-Gutiérrez, Arthur Eumann Mesas, Sergio Núñez de Arenas-Arroyo, Valentina Díaz-Goñi, Celia Álvarez-Bueno, Vicente Martínez-Vizcaíno

**Affiliations:** ^1^Faculty of Nursing, Universidad de Castilla-La Mancha, Cuenca, Spain; ^2^Health and Social Research Center, Universidad de Castilla-La Mancha, Cuenca, Spain; ^3^Center for Biomedical Research Network in Mental Health (CIBERSAM), Instituto de Salud Carlos III, Madrid 28029, Spain; ^4^Research Network on Chronicity, Primary Care and Health Promotion (RICAPPS), Cuenca, Spain; ^5^Faculty of Medicine, Universidad Autónoma de Chile, Talca, Chile

**Keywords:** CES-DdC, children, depression, factor structure, psychometric properties, validation

## Abstract

**Purpose:** The 20-item Center for Epidemiological Studies Depression Scale for Children and Adolescents (CES-DC) is an instrument for screening of depression with good psychometric properties. This study aimed to examine the construct validity in terms of structural and convergent validity, the internal consistency, and the concurrent validity of a shorter 10-item version of this scale.

**Methods:** This was a cross-sectional validation study including 671 schoolchildren aged 9–11, from Cuenca, Spain. Depression was assessed using the 20-item CES-DC scale. We selected the 10 items with the highest factorial loading for a shorter version. Sociodemographic, anthropometric, fitness, and quality of life variables were considered to analyse convergent and discriminant validity.

**Results:** For the structural validity, confirmatory factor analyses revealed a three-factor latent structure for the 20-item CES-DC and a single factor in the 10-item version. Internal consistency measured by Cronbach's *α* and *ω* statistic were 0.85 for 20-item CES-DC and 0.84 for 10-item CES-DC. Intraclass correlation coefficient between the two scales was 0.94. Convergent validity was tested through the correlation coefficients and regression models between both either CES-DC versions with body mass index, waist circumference, fitness, and quality of life measures, which were similar. For the concurrent validity, concordance analysis and the ROC curve showed an equivalent cut-off point for the 10-item CES-DC. The 20-item CES-DC classified a total of 33.1% of the sample as at risk of depression, while the 10-item CES-DC classified 30.4%.

**Conclusion:** This study indicates that both the 20-item and 10-item versions of the CES-DC have good internal consistency and structural validity in schoolchildren. Therefore, this short version can be used as a reliable and valid instrument for screening depression that is less time consuming and easy to use in clinical and scholarly contexts, potentially improving early detection and intervention for depression.

**Trial Registration:** ClinicalTrials.gov identifier: NCT03236337.

## 1. Introduction

Mental health in children and adolescents is an increasingly recognised concern worldwide [1]. Over the past decades, there has been a growing awareness of the importance of mental health in young populations, accompanied by efforts to reduce stigma and improve understanding of mental health problems [2]. This has led to various programmes and policies to improve mental health services, promote early identification, and provide appropriate interventions for young people [1, 3, 4]. Despite these advances, urgent action is needed to address the growing mental health needs of young people [1].

Depression is a mental disorder characterised by persistent sadness, a depressed mood, loss of interest or pleasure, and other symptoms such as irritability that interfere with daily functioning in children and adolescents [5]. It is considered a major cause of disability-adjusted life years worldwide, even in young people [6]. Reliable screening tools are essential for early detection and intervention, which can mitigate the long-term negative impacts of depressive symptoms [7].

Depression is not only among the most prevalent mental disorders [6], but its incidence is increasing in recent years. Thus, a prevalence between 2.5% and 20% of depressive symptoms in children and adolescents [8] was observed in data prior to the COVID-19 pandemic, while this prevalence ranges from between 25% and 30% after the COVID-19 pandemic [9, 10], making depression a major concern for public health [9]. Depressive symptoms in childhood, even when subclinical [8], have been related to an increased risk of future episodes of depression [11]. In addition, these symptoms can affect cognition and learning, social relationships [12, 13], global health [14], and quality of life [15] and may even be detrimental on growth and brain development [16, 17].

Despite the high prevalence of depressive symptoms and their negative impact on short- and long-term health and wellbeing, these symptoms have often been underdiagnosed in children and adolescents [9, 18], and valid and reliable tools are needed to detect people at risk. For this purpose, some of the most common self-administered instruments for screening depressive symptoms are the Children Depression Inventory (CDI) [19], the Children's Depression Scale (CDS) [20], and the Center for Epidemiological Studies Depression Scale for Children (CES-DC) [21]. This latter instrument measures the severity of depression symptoms and can be useful as a screening tool for depression in children and adolescents from 7 to 17 years old. The advantages of the CES-DC include that it is an instrument internationally used, allowing the comparability of its prevalence estimate among countries, that it is easy to use and understandable for schoolchildren, so it can be self-administered [18, 22–25], and that it uses a multidimensional approach to measure symptoms of depression [22].

Adequate content validity has been reported because most items of this scale were originally based on diagnostic criteria for depressive disorders such as the presence of sadness, emptiness, and irritable mood, accompanied by somatic and cognitive changes [26] that affect the individual's capacity [5]. Regarding internal consistency, the scale has shown a Cronbach's *α* values between 0.84 and 0.89 [23, 26], and its validations to other languages displayed Cronbach's alpha*α*'s ranging between 0.80 and 0.90 [18, 22, 27]. Regarding the Spanish validation, this scale also showed good psychometric properties and a Cronbach's *α* of 0.88 [24].

Although several studies have been conducted to validate the CES-DC in different contexts and languages, since cultural and linguistic differences might significantly influence how children and adolescents understand and interpret the scale items, there are several inconsistencies and limitations that need to be considered. In terms of its structural validity, the original Radloff´s scale CES-D [26] and CES-DC [26] resulted in four subscales, including *Depressed Affect*, *Somatic and Retardation*, *Happy*, and *Interpersonal*. While this four-factor structure was confirmed in some studies [22], others did not confirm this structural validity reported by Radloff [28] reporting three [18, 23] or two [29] factors. Similarly, differences have been reported in terms of consistency, which was higher in adolescents than in children [23, 25]. These divergences might be due to specific characteristics of the included samples, such as cultural or age differences. In this sense, some limitations have been mentioned in previous studies, such as relative difficulties in answering some items by children, which might be due to cognitive differences in the understanding of items, or the presence of items and subscales that do not fit with the whole scale [24, 25, 28], indicating the need for further research to explore these discrepancies and to confirm a consistent factor structure.

To make screening of symptoms of depression less time consuming, a 4-item version of the CES-DC (4-item CES-DC) was developed that has shown Cronbach's *α* values between 0.58 and 0.61 [30–32]. Thus, although a short version of the CES-DC might be useful for detecting depressive symptoms in large samples of children and adolescents, more than four items may be needed for an adequate evaluation of this condition [32]. This indicates the need to explore abbreviated versions that preserve the internal consistency and validity of the complete scale.

Therefore, the CES-DC could be a reliable and valid instrument for screening for depressive symptoms in children and adolescents. However, as it is a screening and not a diagnostic instrument, the brevity of completion is a key factor for it to be useful in clinical and schools settings. Furthermore, although the psychometric properties of the Spanish version of the scale have already been evaluated, other aspects of the validation process remain to be investigated, such as the convergent validity of this tool or problems with certain items and subscales. Thus, the aims of this study were to confirm the construct validity in terms of structural and convergent validity and the internal consistency of the Spanish 20-item CES-DC in schoolchildren and to assess whether a short 10-item CES-DC scale has similar psychometric properties and concurrent validity. The findings of this research will not only contribute to the existing literature on the validity of the CES-DC in Spanish-speaking contexts by addressing current gaps but also provide a more efficient tool for the early detection of depression, facilitating timely and effective interventions.

## 2. Methods

This was a cross-sectional validation study of a short version of the CES-DC scale in Spanish 9–12-year-old children. This study was reported according to the COnsensus-based Standards for the selection of health Measurement INstruments (COSMIN) guideline [33, 34] (Table S1).

### 2.1. Participants, Recruitment, and Procedure

We used the baseline measurements of a follow-up study aimed at assessing the influence of daily physical activity on physical and mental health in schoolchildren. All children in 4th, 5th, and 6th grades of primary school in six schools from the province of Cuenca (Spain) were invited to participate in the 1-year follow-up study. The inclusion criterion was that parents or legal guardians provided consent for their children to participate in the follow-up study. The exclusion criteria were medical or psychiatric disorders that would impede the children from accurately answering the questionnaire and poor knowledge of the Spanish language. It was estimated that, considering previous experiences of participation in public Spanish schools, this sample size is considered enough to provide evidence about the validity and internal consistency studies involving patient-reported outcome measures, for which a minimum of 200 individuals has been suggested [35], although a minimum sample size of 300 individuals has been recommended for factor analysis. A total of 745 schoolchildren were ultimately enrolled in the follow-up study. Of these, 74 were removed because of missing data on 20-item CES-DC scale (questionnaires with missing items were removed from the final analysis). As a result, the present analyses were performed with 671 children with complete data (Figure S1). Additionally, no significant differences in age, sex, and socioeconomic status were observed among participants who responded to the questionnaire and were included in the current analyses, compared to those who did not.

The study was approved by the Clinical Research Ethics Committee of the Virgen de la Luz Hospital (2019/PI1519) in Cuenca. Following this, each school's governing board granted permission, and a letter was sent to the parents of all students in each grade, inviting them to a meeting. At the meeting, the study's objectives were explained, and they were given the information sheet and informed consent. Parents or legal guardians were asked to read the information carefully and give written consent if they agreed to their children's participation. Furthermore, the schoolchildren were informed about the details of the study, and they were verbally asked to provide consent prior to the completion of each test. Parents and children were informed that they could revoke the participation agreement at any time. All procedures in this study adhered to the Declaration of Helsinki and its subsequent amendments or comparable ethical standards for human experiments.

No adverse events occurred as a result of the tests. Indeterminate results were considered false positive or false negative and incorporated into the final analysis. Data were collected in each school in October and November 2022 by trained researchers (nurses, physiotherapists, psychologists, teachers, and physical activity experts) following standardised procedures. The research team was available to answer any questions and provide support to any child who felt uncomfortable during the study. All collected data were anonymised. Each participant was assigned a unique code, and personal identifiers were removed from the dataset. Only the research team had access to the code list, which was stored securely and separately from the data.

The previous Spanish version [24] was reviewed. A first pilot of the previously validated Spanish version in children from two schools not included in the follow-up study (*n* = 240) was performed to assess its understandability. As we found that some items were not well understood by the children in our pilot study sample and that these items substantially reduced the internal consistency of the scale (Table S2), the original English version of the CES-DC was translated into Spanish by using the following procedures: First, the original English version of the CES-DC was translated into Spanish by a bilingual English teacher whose mother tongue was Spanish, and then translated into English by another English teacher whose mother tongue was English, and checked for agreement between the two versions [3, 4]; and second, an artificial intelligence tool (DeepL; TechCrunch, USA) was used for the translation and back-translation procedures. The translated and the original versions of the CES-DC are in Tables S3 and S4.

### 2.2. Measures

Socioeconomic status was assessed by using an ad hoc questionnaire filled in by parents, which include the highest level of education in the family (father or mother) and employment situation [36]. A five-category socioeconomic index was calculated from these two variables, and these categories were collapsed into three SES levels: “upper/upper middle”, “middle”, and “lower/lower middle”. The questionnaire also asked about migration status.

Height and weight were measured twice in standardised conditions, and the mean of the two measurements of each variable was used as the final score. Body mass index (BMI) was calculated as weight divided by the square height in meters (kg/m^2^). Then, based on age and sex BMI *z* scores, four weight status categories were calculated according to the International Obesity Task Force BMI cut-off points for thinness, overweight, and obesity [37]. Waist circumference was measured twice at the middle point between the iliac crest and costal margin when the child was upright.

Cardiorespiratory fitness (CRF) was assessed using the 20-m shuttle run test, a valid test to estimate aerobic capacity in children [38]. Maximal oxygen intake (VO^2^ max) was estimated by applying the Leger formula [39].

The CES-DC [21, 23] consists of a self-administered questionnaire of 20 items to assess depressive symptomatology, designed as a Likert-type scale with four response options (0 = *not at all*; 1 = *a little*; 2 = *some*; 3 = *a lot*). The final score of the questionnaire was the sum of the responses obtained on each item (items 4, 8, 12, and 16 are inverse scaled items, so they should be rescaled). The range of the scale is 0–60, and the highest scores indicated the highest level of depression. Although optimal cut-off points vary across samples from 12 to 24, as in our analyses, a cut-off point of 15 has been suggested [30, 40] for potentially clinically significant depressive symptoms.

The KIDSCREEN-27 was used to measure health-related quality of life (HRQoL), Spanish version of which has been shown to have adequate psychometric properties [41]. This questionnaire consisted of a 27-item self-report questionnaire that was used to measure quality of life across five dimensions: physical wellbeing, psychological wellbeing, parent relations and autonomy, social support and peers, and school environment. Higher scores indicate a better quality of life.

### 2.3. Statistical Analyses

For the descriptive analyses, frequency distribution was used for categorical variables and mean and standard deviation (SD) for quantitative variables. Differences in the main characteristics between girls and boys were tested using Student's *t* test for independent samples and chi-square tests.

#### 2.3.1. Construct Validity: Structural Validity

To examine the structural validity underlying both the 20-item and 10-item CES-DC scales, principal component analysis (PCA) was used, using eigenvalues of 1 as criterion to determine the optimal number of factors and Varimax rotation to simplify the interpretation of the factors by minimising the number of variables with high loadings on more than one factor. We used Bartlett's test of sphericity and the Kaiser–Meyer–Olkin (KMO) index to assess the suitability of the factor solution. Kaiser's criterion and the analysis of the scree plot were used to analyse the suitability of the number of factors to extract. The 10 items with the highest factorial loadings, with the number of factors set to 1, were extracted from the PCA analysis. Additionally, Horn's parallel analysis [42] was performed as a sensitivity analysis to determine the optimal number of factors. Factors with eigenvalues greater than the eigenvalues corresponding to those of a matrix of simulated values are considered optimal.

Confirmatory factor analysis (CFA) [43] for 10-item CES-DC was performed with maximum likelihood procedures, the comparative fit index (CFI) [44], the Tucker–Lewis index (TLI) [44], the root mean square error of approximation (RMSEA) and its 90% interval confidence [45], and the standardised root mean square residual (SRMR) [44]. According to [44], fit statistics indicative of a good fit are TLI > 0.95, CFI > 0.95, RMSEA < 0.06, SRMR < 0.08, and models with statistically nonsignificant *χ*^2^. Additionally, multigroup analysis was performed to test the equivalence of the scale across sex and weight status groups.

#### 2.3.2. Internal Consistency

For internal consistency analysis of both the 20-item and 10-item CES-DC scales, Cronbach's *α* has been suggested for unidimensional scales and the McDonald's *ω* (hierarchical) coefficient for multidimensional scales [46]. Thus, we have provided both coefficients. For both indicators, a value of 0.70 or higher indicates acceptable internal consistency [47, 48], while low scores suggest that computing a cumulative score has no sense. In addition, we have provided tables for the two scales including the inter-item correlation, the item-total scale correlation, and the *α*/*ω* if the item is deleted.

#### 2.3.3. Convergent Validity

Because depression in children and adolescents has been associated with obesity [49], low fitness levels [50], and HRQoL [15], we hypothesised that both the 10- and the 20-item CES-DC scales should be related to these variables. Thus, for the analysis of convergent validity of the two tests, the associations between depression score and adiposity, fitness, and HRQoL variables were examined using Pearson's correlation coefficients and linear regression models. The clinical equivalence between the two versions of the CES-DC scale was assessed using Student's t to compare mean differences in BMI, waist circumference, fitness, and HRQoL variables by depression status according to suggested cut-off points (15 and 8, for the 20 and 10 items, respectively). The concordance for detecting children at risk of depression using these cut-off points was assessed using the kappa coefficient.

#### 2.3.4. Construct Validity: Concurrent Validity

Finally, to analyse the concurrent validity and thus confirm the appropriateness of 8 as the cut-off point for the 10-item CES-DC, an receiver operating characteristic (ROC) curve, area under the curve (AUC), specificity, and sensitivity were analysed considering the score of 15 in the 20-item CES-DC scale as the comparison gold standard. An AUC of 0.5 represents a scale with no discriminating ability, while an AUC of 1.0 represents a scale with perfect discrimination [51]. Both sensitivity and specificity should be high for a scale to be accurate (80% or more) [52].

Data analyses were conducted using IBM SPSS Statistics v.28 and Jamovi Desktop Software v.2.3.28, and confirmatory and multigroup analysis were conducted using AMOS Graphics v.28. The level of significance was set at *p* < 0.05.

## 3. Results

The final sample included 671 (339, 50.52% girls) schoolchildren aged 9–11 years (mean = 10.42; SD = 0.94). Participant characteristics are presented in Table 1. The mean scores of the 20-item CES-DC and the 10-item CES-DC were 12.89 (SD = 8.18) and 5.95 (SD = 4.93), respectively. The prevalence of depression risk was 33.4% and 30.4% according to the 20-item CES-DC and the 10-item CES-DC, respectively. Girls showed significantly higher weight status (*p*=0.043) and higher HRQoL in the dimension of school environment (*p*=0.002). Boys showed significantly higher CRF (*p* < 0.001) and physical wellbeing (*p* < 0.001). No significant differences regarding socioeconomic variables were reported across sexes (*p*=0.779).

A total score of 0 (floor effect) was obtained by 2.1% of the participants in the 20-item CES-DC and 9.2% when in the 10-item CES-DC. None of the participants obtained the maximum score in any version (ceiling effect).

### 3.1. Construct Validity: Structural Validity

A three-factor solution for the 20-item CES-DC was found considering both the factor solution of PCA on eigenvalues greater than 1 and scree plot (Table S5 and Figure S2(a)); the values of both tests the KMO (0.915) and the Bartlett sphericity (*X*^2^ = 3403; *gl* = 190; *p* < 0.001) indicated the adequacy of the factorial solution. These three factors explained 43.02% of the variance. The factor loadings of the 20-item CES-DC are shown in Table 2. All items had a factor loading above 0.30, with a range of 0.324–0.715, with item 10 (*I felt scared*) having the lowest factor loading.

The 10 more weighted items in were selected for the 10-item CES-DC. The factor solution of the 10-item CES-DC was assessed using PCA and showed a single latent factor solution based on eigenvalues greater than 1. The scree plot showed a single suitable factor solution for the 10-item CES-DC (Table S5 and Figure S2(b)). The result of the KMO test was 0.903, and the Bartlett sphericity was significant (*X*^2^ = 1999; *gl* = 45; *p* < 0.001). This factor explained 42.67% of the variance. Factor loadings are shown in Table 2, and all items had a factor loading above 0.30.

As a sensitivity analysis, Horn's parallel was performed. A graphical representation of the parallel analysis result is presented in Figure S3. The eigenvalues of the principal component solution suggested one factor for the 10-item CES-DC and two factors for the 20-item CES-DC. Despite the difference found for the 20-item CES-DC, items corresponding to factor 1 aligned with factors 1 and 2 of the PCA (*depressive affect* and *somatic retardation*), while factor 2 aligned with factor 3 (*happy*) of the PCA. This justifies the structure of the 10-item CES-DC, which includes only items from factors 1 and 2 of the PCA.

**Figure 1:**
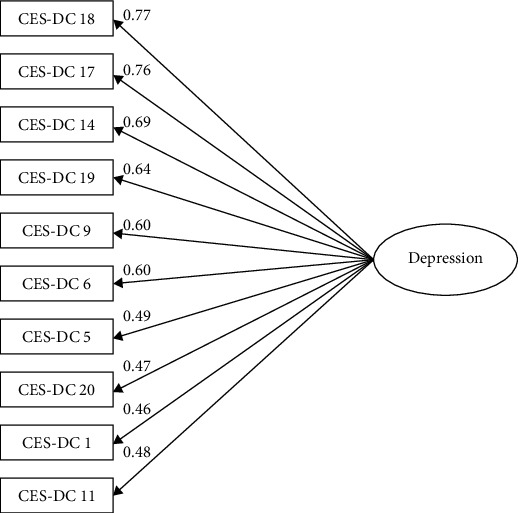
*χ* ^2^ = 147; df = 35; *p* < 0.001; CFI = 0.94, TLI = 0.93, RMSEA (IC 90%) = 0.07 (0.06, 0.08), and SRMR = 0.04.

The single-factor model proposed for the CFA of the 10-item CES-DC showed an adequate fit (*χ*2 = 147; *df* = 35; *p* < 0.001; CFI = 0.94, TLI = 0.93, RMSEA (IC 90%) = 0.07 (0.06, 0.08), and SRMR = 0.04); see Figure 1. Multigroup analyses were conducted by sex and weight status [37]. The model comparison analyses show that the model was equivalent by sex (*p*=0.469) and weight status (*p*=0.09) with significant differences of measurement weights on item 18 (Figures S4 and S5).

### 3.2. Internal Consistency

Consistency measured by McDonald's *ω* statistics was 0.850 for the 20-item CES-DC, 0.827 for the depressed affect subscale, 0.703 for the somatic and retardation subscale, and 0.602 for the happiness subscale. McDonald's *ω* for the 20-item CES-DC improved after removal of items 4 (*ω* of 0.856 after the item was removed) and 8 (*ω* of 0.856 after the item was removed) and for the somatic and retardation subscale improved after removal of item 3 (*ω* of 0.832 after the item was removed). McDonald's *ω* was 0.840 for the 10-item CES-DC (Table S6).

For Cronbach's *α*, it was 0.852 for the 20-item CES-DC, 0.828 for the depressed affect subscale, 0.702 for the somatic and retardation subscale, and 0.608 for the happiness subscale. Cronbach's *α* for the 20-item CES-DC improved after removal of items 4 (*ω* of 0.857 after the item was removed) and 8 (*ω* of 0.856 after the item was removed) and for the somatic and retardation subscale improved after removal of item 3 (*ω* of 0.833 after the item was removed). Cronbach's *α* of the 10-item CES-DC was 0.841 and did not increase after eliminating any of the items (Table S6).

The corrected item-total correlation coefficients ranged from 0.346 to 0.664 (Table S6). All these values were above the minimum corrected item-total correlation of 0.20, which has been recommended for including an item in a scale [53]. Table S7 illustrates the pattern of inter-item correlations between all items. All items of the subscales of the 20-item CES-DC scale and the 10-item CES-DC scale correlated with each other.

### 3.3. Construct Validity: Convergent Validity

To assess the convergent validity of both versions of the CES-DC, we examined the Pearson's correlation coefficients and linear regression models between the CES-DC scores (both 20-item and 10-item) and BMI, waist circumference, and HRQoL global and dimensions (Table 3 and Table S8). The total scores of both the 10-item and the 20-item scales of the CES-DC were positively and significantly associated with BMI and with waist circumference (*p* < 0.05). In contrast, higher scores on the two versions of the CES-DC had a negative and significant association with global quality of life and the quality-of-life dimensions physical wellbeing, psychological wellbeing, autonomy and parent relations, peer relations, and school environment (*p* < 0.001). Both the 20-item CES-DC scale and the 10-item CES-DC scale showed similar patterns of associations, demonstrating that they measured the same underlying construct.

Children classified as depressed by both the 20-item and 10-item scales had higher BMI and waist circumference and lower CRF and quality of life in all dimensions compared to nondepressed children (Table 4). These analyses showed that both versions of the CES-DC can effectively discriminate between nondepressed and depressed children, supporting the validity of the 10-item version as a shorter alternative to the 20-item version.

### 3.4. Concurrent Validity

For the 20-item CES-DC, the cut-off point for detecting children and adolescents at risk of depression was set as greater than 15 [23]. According to these points, the 20-item CES-DC classified a total of 33.1% of the sample as at risk of depression. To establish an equivalent cut-off point for the 10-item CES-DC, an ROC curve analysis was performed. For a 7.5 cut-off point (greater than 7, because this scale provides integers numbers), the AUC was 0.97 indicating an excellent discriminative ability of the 10-item scale to distinguish between individuals with and without risk of depression. Sensitivity and specificity values were 93.8% (percentage of children at risk of depression who were correctly classified) and 84.2% (percentage of children not at risk of depression who were correctly classified), respectively (Figure S6), rating a 30.4% as at risk of depression (Table 5). A sensitivity analysis was conducted by setting a cut-off point higher than 6, which revealed that 37.0% of children were at risk of depression according to the 10-item CES-DC (Table S9).

## 4. Discussion

The psychometric properties of the CES-DC scale have been well consistently proven in several countries and in several languages. However, a scale of 20 items is still too long to be self-administered by children. Our study, in addition to confirming that the Spanish version of the CES-DC has an acceptable structural and convergent validity and internal consistency, presents these psychometric properties for a shortened 10-item scale. Results of our study show that the 10-item CES-DC has adequate psychometrical properties and is equivalent to the 20-item CES-DC version in children aged 9–11 years old, with a substantial agreement between both versions for classifying children as at risk of depression (kappa = 0.78). Additionally, while a three-factor latent structure underlies the 20-item CES-DC, a single factor underlies the 10-item version.

In our study, the internal consistency values of the 20-item version were high with a Cronbach's *α* and an *ω* coefficient of 0.85 showing a strong consistency. The shortened 10-item version showed similar consistency values (0.84) with the 20-item Spanish version and with those reported in the original full versions of the scale and previous validations, between 0.77 and 0.91 [23, 26, 27, 30], and greater than those reported for the 4-item version proposed by Fendrich, Weissman, and Warner [30]. These results were similar to the previous Spanish validation of the CES-DC; however, in that version, authors found that item 7 (*I felt like I was too tired to do things*) had a factor loading below 0.30; and consequently, it was removed from the scale. In contrast, in this version, item 7 had an adequate factor loading in the expected factor (0.679 in factor 2: *Somatic and Retardation*). These differences might be due to the retranslation process, in which some items showed that they were difficult to understand for the children evaluated in the pilot phase of the study. Those items were identified and rewritten to facilitate their understanding. Factor loadings in the 10-item version ranged between 0.540 and 0.772, showing adequate functioning.

The structure of the CES-DC is a debatable issue in the literature. The original proposed model with a four-factor (dimensions) structure [26, 27] was replicated in the Spanish previous validation [24]. However, in our results, this four dimensions version was not replicated. According to our data, although eigenvalues might suggest a four-factor structure, items 15 and 19 did not fit together on an *interpersonal* fourth dimension. This result is congruent with other studies that did not find the *interpersonal* dimension [23, 27]. Moreover, other authors have suggested the need for at least three items to form a dimension [54]. Cultural and linguistic differences may have significantly influenced how children understood and interpreted the scale items. The Spanish version of the CES-DC used in our study may have subtle differences in item wording or cultural context that affect how children perceive and respond to the questions. This may have led to variations in the factor structure compared to versions validated in other languages and cultural contexts. The discrepancies observed underscore the importance of conducting rigorous validation studies in diverse populations. Regarding the 10-item CES-DC version, an underlying factor was observed. A simpler, unidimensional scale might be easier for children to complete and understand, fulfilling the same screening purpose as the 20-item version. In addition, it can be administered more quickly without causing fatigue or cognitive overload for children, making it useful in clinical and school settings.

Regarding cut-off points, several studies have suggested a cut-off point of more than 15 [21, 23] for identifying children and adolescents at risk of depression. In this study, this cut-off point of 15 was surpassed for one-third of the children. Although these figures of children at risk of depression may appear too high, they are similar to those reported by recent prevalence studies [10] that estimated a depressive symptom prevalence of 34% and major depressive disorder and dysthymia of 8% and 4%, respectively [55]. Other authors have proposed the setting of higher cut-off points [26, 27] or the establishment of a higher cut-off point to differentiate moderate from severe depression. For the 10-item version, an equivalent cut-off point of 7.5 was tested, showing similar discriminant capacity to the 20-item version. This threshold is essential to ensure that the screening tool is both sensitive and specific enough to identify children who may need further assessment or intervention. In conclusion, the identified cut-off point offers a practical means of screening for depression risk. However, its optimal use requires careful consideration and further validation. For these reasons, further research comparing different cut-off points to a clinical diagnosis by an expert in major depression would serve to complete the process of validating the scale for clinical use.

Our data, congruently with previous studies [49], show that children at high risk of depression had higher BMI and waist circumference, which might be due to overweight concerns and weight stigma [56] or to inflammatory processes, common in obesity, that affect brain and overall health [57]. Higher physical fitness is associated with better psychological wellbeing, potentially due to the positive effects of physical activity on neuroplasticity and mood-related neurotransmitters [50], and also higher HRQoL is associated with better mental health, similar to other studies [15]. These results support the convergent validity of both versions of the scale and underline the importance of promoting healthy school environments with integrated interventions that address both children's mental and physical health.

These results should be interpreted with caution given the limitations of this study. First, as a cross-sectional study, these results do not establish the predictive validity of the scales, which implies that we cannot infer causality or long-term outcomes; therefore, longitudinal studies are needed to assess the capacity of the CES-DC to predict future depressive symptoms or episodes. Second, the absence of a gold standard instrument to measure depression for comparison limits the ability to validate the CES-DC against a well-established diagnostic tool. Third, while the CES-DC is designed to measure the risk of depression in both children and adolescents, our sample only included children aged 9–12. Therefore, these findings may not be generalisable to older adolescents. Additional studies should be conducted to confirm these results in a broader age range, including adolescents. Fourth, shorter versions can probably be developed in the future for routine use in the clinical assessment of adolescents; however, as this is the minimum number of items that could most efficiently represent the symptoms of depression in this age group and at the same time remain a single dimension, we believe that this first shorter approach to the scale may have sufficient face validity and be a simple and easy to use scale. Finally, this is the first validation of this shorter version, and more research is needed to confirm its reliability and validity in different populations and settings.

In conclusion, the results of this study support that the 20-item CES-DC and its short 10-item version have adequate structural and convergent validity and internal consistency for Spanish children. Moreover, both versions of the scale are equivalent for screening of depression in a sample of children, and the short version of the instrument provides a short, simple instrument that requires little time for children to complete in an autonomous way. For that reason, the 10-item CES-DC may be a suitable instrument for screening depression in scholarly and clinical studies.

## Figures and Tables

**Table 1:** Sociodemographic characteristics of the sample.

Baseline characteristic	Total *N* = 671	Boys *n* = 332	Girls *n* = 339	*p* value
Age (years)	10.42 (0.94)	10.39 (0.93)	10.45 (0.96)	0.394
Weight status (BMI)				
Low	59 (8.8%)	21 (6.3%)	38 (11.2%)	—
Normal	406 (60.5%)	203 (61.1%)	193 (56.9%)	0.043
Overweight/Obesity	206 (30.7%)	98 (29.5%)	108 (31.9%)	—
Waist circumference (cm)	65.38 (10.83)	66.16 (11.39)	64.61 (10.22)	0.064
Cardiorespiratory fitness (VO^2^ max; ml/kg/min)	45.59 (4.48)	46.98 (4.90)	44.20 (3.50)	<0.001
Socioeconomic status				
Lower/lower middle	105 (15.7%)	50 (15.1%)	55 (16.2%)	—
Middle	163 (24.3%)	81 (24.4%)	82 (24.2%)	0.779
Upper/upper middle	57 (8.5%)	25 (7.5%)	32 (9.4%)	—
Migratory status				
Yes	34 (5.1%)	13 (3.9%)	17 (5.0%)	0.756
No	443 (66.0%)	152 (45.8%)	162 (47.8%)	—
Depression (20-item CES-DC score)	12.89 (8.18)	13.25 (8.31)	12.56 (8.05)	0.244
Depression status (20-item CES-DC)^a^				
Nondepressed	449 (66.9%)	213 (64.2%)	236 (69.6%)	0.133
Depressed	222 (33.1%)	119 (35.8%)	103 (30.4%)	—
Depression (10-item CES-DC score)	5.95 (4.93)	6.13 (5.17)	5.78 (4.69)	0.078
Depression status (10-item CES-DC)^b^				
Nondepressed	467 (69.6%)	222 (66.9%)	245 (72.3%)	0.128
Depressed	204 (30.4%)	110 (33.1%)	94 (27.7%)	—
HRQoL (score)	54.57 (9.83)	54.65 (9.33)	54.48 (10.31)	0.825
Physical wellbeing	52.11 (9.39)	53.49 (9.26)	50.74 (9.34)	<0.001
Psychological wellbeing	53.67 (9.72)	53.81 (9.34)	53.52 (10.08)	0.699
Parents relations and autonomy	52.23 (10.03)	52.03 (9.49)	52.42 (10.54)	0.621
Social support and peers	55.11 (9.21)	54.98 (9.20)	55.24 (9.25)	0.717
School environment	57.64 (10.15)	56.40 (10.43)	58.85 (9.72)	0.002

*Note:* Data are presented as *n* and % except for age and depression score, waist circumference, cardiorespiratory fitness, and quality of life presented in M and SD. A total score of 0 (floor effect) was obtained by 2.1% of the participants in the 20-item CES-DC and 9.2% when in the 10-item CES-DC. None of the participants obtained the maximum score in any version (ceiling effect).

Abbreviations: BMI, body mass index; HRQoL, health-related quality of life.

^a^Cut-off point for the 20-item CES-DC was set at 15.

^b^Cut-off point for the 10-item CES-DC was set at 8. It is equivalent to the 20-item CES-DC cut-off point that was established from a ROC curve analysis (AUC = 0.97).

**Table 2:** Principal component analysis of 20-item and 10-item CES-DC.

CES-DC 20-item	Factor loading		
	1	2	3
Factor 1. Depressed affect			
14. I felt lonely, like I didn't have any friends.	0.715	—	—
19. I felt people didn't like me.	0.707	—	—
18. I felt sad.	0.673	—	—
17. I felt like crying.	0.654	—	—
6. I felt down and unhappy.	0.613	—	—
15. I felt like kids I know were not friendly or that they didn't want to be with me.	0.569	—	—
3. I wasn't able to feel happy, even when my family or friends tried to help me feel better.	0.449	—	—
9. I felt like things I did before didn't work out right.	0.421	—	—
10. I felt scared.	0.324	—	—
Factor 2. Somatic and retardation			
7. I felt like I was too tired to do things.	—	0.679	—
5. I felt like I couldn't pay attention to what I was doing.	—	0.619	—
20. It was hard to get started doing things.	—	0.574	—
11. I didn't sleep as well as I usually sleep.	—	0.536	—
2. I did not feel like eating, I wasn't very hungry.	—	0.491	—
1. I was bothered by things that usually don't bother me.	—	0.482	—
13. I was more quiet than usual.	—	0.407	—
Factor 3. Happy			
8. I felt like something good was going to happen. (R)	—	—	0.713
12. I was happy. (R)	—	—	0.681
16. I had a good time. (R)	—	—	0.681
4. I felt like I was just as good as other kids. (R)	—	—	0.609

**CES-DC 10-item**			**1**

18. I felt sad.			0.772
17. I felt like crying.			0.767
14. I felt lonely, like I didn't have any friends.			0.724
19. I felt people didn't like me.			0.678
9. I felt like things I did before didn't work out right.			0.667
6. I felt down and unhappy.			0.659
5. I felt like I couldn't pay attention to what I was doing.			0.575
20. It was hard to get started doing things.			0.548
1. I was bothered by things that usually don't bother me.			0.543
11. I didn't sleep as well as I usually sleep.			0.540

*Note: N* = 671. The extraction method was principal axis factoring with a maximum likelihood (Varimax) rotation. Reverse-scored items are denoted with an (R).

**Table 3:** Pearson's correlations among 20-item CES-DC and 10-item CES-DC and measures of anthropometry, fitness, and quality of life.

Variable	1	2	3	4	5	6	7	8	9	10
1. CES-DC 20	—									
2. CES-DC 10	0.94^*∗∗*^	—								
3. BMI	0.11^*∗∗*^	0.12^*∗∗*^	—							
4. Waist circumference	0.09^*∗*^	0.09^*∗*^	0.92^*∗∗*^	—						
5. CRF	−0.11^*∗∗*^	−0.09^*∗*^	−0.46^*∗∗*^	−0.46^*∗∗*^	—					
6. HRQoL	−0.61^*∗∗*^	−0.53^*∗∗*^	−0.12^*∗∗*^	−0.12^*∗∗*^	0.18^*∗∗*^	—				
7. Physical wellbeing	−0.27^*∗∗*^	−0.20^*∗∗*^	−0.16^*∗∗*^	−0.16^*∗∗*^	0.31^*∗∗*^	0.63^*∗∗*^	—			
8. Psychological wellbeing	−0.64^*∗∗*^	−0.58^*∗∗*^	−0.08^*∗*^	−0.08^*∗∗*^	0.16^*∗∗*^	0.74^*∗∗*^	0.42^*∗∗*^	—		
9. Autonomy and parent relation	−0.46^*∗∗*^	−0.39^*∗∗*^	−0.03	−0.03	0.09^*∗∗*^	0.68^*∗∗*^	0.39^*∗∗*^	0.49^*∗∗*^	—	
10. Peer relations	−0.37^*∗∗*^	−0.33^*∗∗*^	−0.07	−0.07	0.13^*∗∗*^	0.56^*∗∗*^	0.43^*∗∗*^	0.45^*∗∗*^	0.44^*∗∗*^	—
11. School environment	−0.54^*∗∗*^	−0.40^*∗∗*^	−0.13^*∗∗*^	−0.13^*∗∗*^	0.05	0.62^*∗∗*^	0.37^*∗∗*^	0.47^*∗∗*^	0.38^*∗∗*^	0.43^*∗∗*^

*Note: N* = 671.

Abbreviations: BMI, body mass index; CRF, cardiorespiratory index; HRQoL, health-related quality of life.

*⁣* ^*∗*^ *p* < 0.05; *⁣*^*∗∗*^*p* < 0.01.

**Table 4:** Difference in mean of body composition, fitness, and health-related quality of life by depression status in children using 20-item CES-DC and 10-item CES-CD.

Measure	Non-depressed		Depressed		*p* value
	Mean	SD	Mean	SD	
20-item CES-DC^a^					
BMI	18.52	3.49	19.26	4.23	0.017
Waist circumference	64.77	10.48	66.59	11.44	0.020
CRF	45.95	4.56	44.86	4.21	0.004
HRQoL	57.80	9.01	47.92	7.92	<0.001
Physical wellbeing	53.49	8.78	49.29	9.99	<0.001
Psychological wellbeing	57.07	8.58	46.72	8.05	<0.001
Autonomy and parent relation	54.78	9.13	47.05	9.78	<0.001
Peer relations	56.99	7.84	51.27	10.57	<0.001
School environment	60.02	9.06	52.80	10.52	<0.001
10-item CES-DC^b^					
BMI	18.47	3.51	19.43	4.21	0.003
Waist circumference	64.67	10.55	67.00	11.31	0.010
CRF	45.87	4.51	44.96	4.34	0.009
HRQoL	57.15	9.34	48.62	8.22	<0.001
Physical wellbeing	53.05	9.05	49.96	9.83	<0.001
Psychological wellbeing	56.50	8.90	47.15	8.27	<0.001
Autonomy and parent relation	53.95	9.46	48.28	10.20	<0.001
Peer relations	56.51	8.25	51.91	10.45	<0.001
School environment	59.70	9.33	52.92	10.39	<0.001

*Note: n* = 671.

Abbreviations: BMI, body mass index; CRF, cardiorespiratory index; HRQoL, health-related quality of life.

^a^Cut-off for the 20-item CES-DC-20 was set at 15.

^b^Cut-off for the 10-item CES-DC was set at 8.

**Table 5:** Concordance between 20-item CES-DC and 10-item CES-DC for the screening of depression in children.

20-item CES-DC^a^	10-item CES-DC^b^		Total
	Nondepressed	Depressed	
Nondepressed	426 (63.3%)	23 (3.4%)	449 (66.9%)
Depressed	41 (6.1%)	181 (27%)	222 (33.1%)
Total	467 (69.6%)	204 (30.4%)	671 (100%)

*Note:* Data are presented as *n* and %. Cohen's kappa coefficient = 0.78.

^a^Cut-off for the 20-item CES-DC-20 was set at 15.

^b^Cut-off for the 10-item CES-DC was set at 8.

## Data Availability

De-identified data are available at Visier-Alfonso, M. E. (2023). CES-DC validation. https://doi.org/10.17605/OSF.IO/8UYV9

## Supporting Information

Supporting Information Table S1. COSMIN reporting recommendations for studies on measurement properties. Table S2. Factor loadings and internal consistency of the pilot of the previous Spanish version of CES-DC 20-item. Table S3. Final items of 20-item CES-DC Spanish version. Table S4. Original CES-DC scale. Table S5. Comparative analysis of the structural validity of the 20-item CES-DC and the 10-item CES-DC scales. Table S6. Corrected item-total correlation and Cronbach's *α* and McDonald's *ω* if item deleted. Table S7. CES-DC scale inter-item correlation. Table S8. Linear regression models among 20-item CES-DC and 10-item CES-DC and measures of anthropometry, fitness, and quality of life. Table S9. Concordance between 20-item CES-DC and 10-item CES-DC for the screening of depression in children with cut-off for the 10-item CES-DC set at 7. Figure S1. Diagram flow of the study participants in the current study. Figure S2. Sedimentation graph of factor components of 20-item CES-DC and 10 item CES-DC. Figure S3. Parallel analysis of 20-item CES-DC and 10-item CES-DC. Figure S4. Factor loading and goodness of fit of CES-DC 10 by sex. Figure S5. Factor loading and goodness of fit of CES-DC 10 by body mass index cut-offs. Figure S6. Receiver operating characteristics (ROC) curve and cut-off plot for the 10-item CES-DC in comparison with 20-item CES-DC.
