# Supplementary material for: Internal Consistency and Validity of a Short Spanish Version (10-Items) of the Center for Epidemiological Studies Depression Scale for Children and Adolescents (CES-DC)
Source: Depress Anxiety. 2024 Sep 25;2024:5409747. doi: 10.1155/2024/5409747 (PMC11918615; doi:10.1155/2024/5409747)
Supplement: Supporting Information — Table S1. COSMIN reporting recommendations for studies on measurement properties. Table S2. Factor loadings and internal consistency of the pilot of the previous Spanish version of CES-DC 20-item. Table S3. Final items of 20-item CES-DC Spanish version. Table S4. Original CES-DC scale. Table S5. Comparative analysis of the structural validity of the 20-item CES-DC and the 10-item CES-DC scales. Table S6. Corrected item-total correlation and Cronbach's α and McDonald's ω if item deleted. Table S7. CES-DC scale inter-item correlation. Table S8. Linear regression models among 20-item CES-DC and 10-item CES-DC and measures of anthropometry, fitness, and quality of life. Table S9. Concordance between 20-item CES-DC and 10-item CES-DC for the screening of depression in children with cut-off for the 10-item CES-DC set at 7. Figure S1. Diagram flow of the study participants in the current study. Figure S2. Sedimentation graph of factor components of 20-item CES-DC and 10 item CES-DC. Figure S3. Parallel analysis of 20-item CES-DC and 10-item CES-DC. Figure S4. Factor loading and goodness of fit of CES-DC 10 by sex. Figure S5. Factor loading and goodness of fit of CES-DC 10 by body mass index cut-offs. Figure S6. Receiver operating characteristics (ROC) curve and cut-off plot for the 10-item CES-DC in comparison with 20-item CES-DC. [file 5409747.f1.docx]

Table S1. COSMIN Reporting Recommendations for Studies on Measurement Properties.

| **Item Number** | **Item Name** | **Item Description** | **Reporting Location(s)** |
| --- | --- | --- | --- |
| **Reporting section: Title** | | | |
| T1 | Patient Reported Outcome Measure (PROM) | The name of the PROM instrument(s) (and version if relevant) being studied | Page 1 |
| T1 | Measurement Property (MP) | What MPs are being studied or more generally, that MPs are being studied (if there are many properties being investigated for example). | Page 1 |
| T3 | Study Sample | General description of relevant study sample characteristics (e.g., condition of interest, language) and also any intervention or exposure (e.g., treatments) if applicable. | Page 1 |
| **Reporting section: Abstract** | | | |
| A1 | PROM | The name of the PROM instrument(s) (and version if relevant) being studied (i.e., the SF-36 or SF-12; language version) or if it concerns an item bank (e.g., PROMIS instruments). The type of instrument (e.g., a self-reported questionnaire or interview). | Page 3 |
| A2 | Measurement Property | What MPs are being studied or more generally, that MPs are being studied (if there are many properties being investigated, for example) | Page 3 |
| A3 | Design | The type of study being used to test the properties (e.g., test-retest design, longitudinal study, cohort, cross sectional, case series, randomized etc.). Other details of the study design if relevant (intervention/exposure, description of comparison instruments,  outcomes other than PROMs). | Page 3 |
| A4 | Sample | General description of relevant study sample characteristics (e.g., condition of interest, geographic location, language, other relevant demographic and baseline  characteristics). | Page 3 |
| A5 | Methods | A brief description of the methods for investigating each MP including  statistical analyses. | Page 3 |
| A6 | Results | The main results for all MPs investigated reporting statistics for each result with measures of precision where appropriate. | Page 3 |
| A7 | Discussion/Conclusions | A brief description of the results in the context of existing evidence, main strengths and drawbacks and the need for future research on the  PROM(s) investigated. | Page 3 |
| **Reporting section: Introduction** | | | |
| I1 | Name and describe the PROM of interest | Specify the name, type, language, and version of the PROM being investigated and how it was developed. Describe the construct the PROM aims to measure and its subscales; describe the structure of the PROM (e.g., the number of factors, the number of items, scoring algorithm); describe relevant instructions (like time period), and number or type of response categories. State whether the PROM is based on a reflective or formative model. | Pages 5 and 6 |
| I2 | Target population | Describe the specific target population that the PROM was designed for. The authors need to provide the appropriate and necessary characteristics of this population. | Pages 4, 5 and 6 |
| I3 | Citation for the original development of the PROM | The citation for the original development paper(s) should be provided and other highly relevant citations related to the quality of the specific PROM under investigation. | Pages 5 and 6 |
| I4 | State of knowledge and rationale | A description of the current scientific knowledge (what is known) regarding the MPs of the PROM under investigation. The authors should provide a literature review or refer to a recent review of all existing evidence of the specific version (e.g., language, short 6 form) of the PROM and explain why the new study is necessary and important. The rationale for the current proposed study should be given. | Pages 4, 5 and 6 |
| I5 | Definitions | Specialized terms should be defined or explained. | Pages 4 and 5 |
| I6 | Objectives or Hypotheses | State the specific objectives of the research and hypotheses related to the specific PROM under investigation. | Page 6 |
| **Reporting section: General Methods** | | | |
| GM1 | Study design | State the key elements of the study design. | Page 6 |
| GM2 | Participants | State how the participants were chosen; the inclusion and exclusion criteria (e.g., if PROM for a specific condition, then the eligibility and selection criteria should reflect this). | Pages 6 and 7 |
| GM3 | PROM administration | An explicit description of how and when the PROM(s) were administered (e.g., in what setting) including data collection devices/system used (e.g., paper based, electronic administration/ePRO) should be provided. | Pages 8 and 9 |
| GM4 | Data collection procedures | Provide information about other data collection, exposure methods (e.g., allocation to interventions) and time points/follow-up points. | Pages 7, 8 and 9 |
| GM5 | Power/sample size calculation | Provide a power calculation for all MP analyses. Alternatively, if a rule of thumb is used, state it and the source/citation. | NR |
| GM6 | Statistical analyses | Statistical analysis and tests corresponding to all hypotheses or objectives for all MPs should be reported. Where appropriate, a cut-off for statistical significance should be reported (e.g., p-value less than 0.05). A description of all statistics to be used to estimate the magnitude and direction of effect should also be reported, together with measures of variability or precision. Report statistical packages used. | Pages 9, 10 and 11 |
| GM7 | Missing data | State approaches or plan for dealing with missing data. | Page 7 |
| GM8 | Post-hoc analysis | The report should specify analyses that used data after the data collection period concluded (i.e., if the analyses were post-hoc; secondary data analyses) and describe the rationale for any post-hoc analyses. | NR |
| **Reporting section: General Results** | | | |
| GR1 | Missing data | The amount and reasons for missing data should be explained for all analyses for all PROMs (or other outcome measurement instruments) an relevant groups. | Page 7 |
| GR2 | Participant/patient characteristics | The study patients’ characteristics should be described, including baseline PROM scores. | Page 11 and Table 1 |
| GR3 | Sample size | If one study contained analyses using different sample sizes, the authors should report the sample size for each analysis. | Supplementary material Figure S4 and Figure S5 |
| **Reporting section: Discussion** | | | |
| D1 | MP evidence | Per measurement property the authors should compare the result to the criteria for good measurement properties and determine if the specific MP is sufficient or not. Note: This information may also appear in the results section in greater detail in a table for example. | Pages 17, 18 and 19 |
| D2 | Practice relevance | The authors need to discuss the practical relevance of the findings. | Pages 17, 18 and 19 |
| D3 | Strengths and limitations | Strengths and limitations of the study should be discussed. For example, discuss if there were any significant potential biases in the study which could have impacted the results. | Page 19 |
| D4 | Generalizability | Generalizability issues related to the PROM results should be discussed. For example, discuss if the results could be generalized to other populations given the sample studied. | Pages 17, 18 and 19 |
| D5 | Instrument changes | Discuss the need for modification to the existing PROM or new PROM development. If you conclude that one of the measurement properties is insufficient, you could suggest some modification, or if it is really poor, you could suggest stopping use of the PROM (in the specific population or in general). | NR |
| D6 | Future research | Report specifically the type of research needed to answer new questions arising out of these findings for the particular MP and PROM investigated. | Page 19 |
| **Report section: Conclusion** | | | |
| C1 | Conclusions | State the overall conclusions for each MP and of the use PROM investigated. | Page 20 |
| **Report section: Other information** | | | |
| O1 | Conflicts of interest | State any relevant conflict of interest related to the PROM under investigation (e.g., an author being the PROM developer, funding body etc). | Page 1 |

Table S2. Factor loadings and internal consistency of the pilot of the previous Spanish version of CES-DC 20-item.

| Item | Factor loadings | Corrected item total correlation | Cronbach’s alpha if item deleted |
| --- | --- | --- | --- |
| 1 | 0.579 | 0.420 | 0.767 |
| 2 | 0.405 | 0.209 | 0.777 |
| 3 | 0.580 | 0.362 | 0.768 |
| 4 (R) | -0.119 | 0.105 | 0.817 |
| 5 | 0.549 | 0.314 | 0.768 |
| 6 | 0.793 | 0.617 | 0.760 |
| 7 | -0.090 | 0.132 | 0.812 |
| 8 (R) | 0.062 | 0.166 | 0.799 |
| 9 | 0.622 | 0.426 | 0.765 |
| 10 | 0.501 | 0.321 | 0.774 |
| 11 | 0.399 | 0.296 | 0.778 |
| 12 (R) | 0.571 | 0.372 | 0.767 |
| 13 | 0.381 | 0.235 | 0.779 |
| 14 | 0.634 | 0.460 | 0.766 |
| 15 | 0.527 | 0.334 | 0.772 |
| 16 (R) | 0.563 | 0.354 | 0.767 |
| 17 | 0.777 | 0.624 | 0.754 |
| 18 | 0.688 | 0.540 | 0.761 |
| 19 | 0.711 | 0.516 | 0.762 |
| 20 | 0.498 | 0.269 | 0.774 |
| Cronbach's alpha: 0.784 | | | |

Table S3. Final items of 20-item CES-DC Spanish version.

| **DURANTE LA SEMANA PASADA** |
| --- |
| *1. Me molestaban cosas que no solían molestarme |
| 2. No me apetecía comer, no tenía hambre |
| 3. No he sido capaz de sentirme feliz, incluso cuando mi familia o amigos han intentado ayudarme |
| 4. He sentido que soy tan bueno como los demás niños y niñas (R) |
| *5. Tenía problemas para concentrarme en lo que estaba haciendo |
| *6. Me he sentido desanimado e infeliz |
| 7. Me he sentido demasiado cansado para hacer cosas |
| 8. He sentido que iban a pasar cosas buenas (R) |
| *9. He sentido que las cosas que hacía no me salían bien |
| 10. Me he sentido asustado |
| *11. No he dormido bien |
| 12. He estado contento (R) |
| 13. He estado más callado que de habitual |
| *14. Me he sentido solo |
| 15. He sentido que los niños y niñas que conozco han sido poco amables o no querían estar conmigo |
| 16. Lo he pasado bien (R) |
| *17. He tenido ganas de llorar |
| *18. Me he sentido triste |
| *19. Me parecía que no le gustaba a la gente |
| *20. Me ha costado empezar a hacer cosas |

*Note:* *indicate items selected for the 10-item CES-DC version. (R) indicate reversed items

Table S4. Original CES-DC scale.

**Center for Epidemiological Studies Depression Scale for Children (CES-DC)**

| **DURING THE PAST WEEK** | **Not At All** | **A Little** | **Some** | **A Lot** |
| --- | --- | --- | --- | --- |
| 1. I was bothered by things that usually don’t bother me. | _____ | _____ | _____ | _____ |
| 2. I did not feel like eating, I wasn’t very hungry. | _____ |  |  |  |
| 3. I wasn’t able to feel happy, even when my family or friends tried to help me feel better. | _____ | _____ | _____ | _____ |
| 4. I felt like I was just as good as other kids. | _____ | _____ | _____ | _____ |
| 5. I felt like I couldn’t pay attention to what I was doing. | _____ | _____ | _____ | _____ |

| **DURING THE PAST WEEK** | **Not At All** | **A Little** | **Some** | **A Lot** |
| --- | --- | --- | --- | --- |
| 6. I felt down and unhappy. | _____ | _____ | _____ | _____ |
| 7. I felt like I was too tired to do things. | _____ | _____ | _____ | _____ |
| 8. I felt like something good was going to happen. | _____ | _____ | _____ | _____ |
| 9. I felt like things I did before didn’t work out right. | _____ | _____ | _____ | _____ |
| 10. I felt scared. | _____ | _____ | _____ | _____ |

| **DURING THE PAST WEEK** | **Not At All** | **A Little** | **Some** | **A Lot** |
| --- | --- | --- | --- | --- |
| 11. I didn’t sleep as well as I usually sleep. | _____ | _____ | _____ | _____ |
| 12. I was happy. | _____ | _____ | _____ | _____ |
| 13. I was more quiet than usual. | _____ | _____ | _____ | _____ |
| 14. I felt lonely, like I didn’t have any friends. | _____ | _____ | _____ | _____ |
| 15. I felt like kids I know were not friendly or that they didn’t want to be with me. | _____ | _____ | _____ | _____ |

| **DURING THE PAST WEEK** | **Not At All** | **A Little** | **Some** | **A Lot** |
| --- | --- | --- | --- | --- |
| 16. I had a good time. | _____ | _____ | _____ | _____ |
| 17. I felt like crying. | _____ | _____ | _____ | _____ |
| 18. I felt sad. | _____ | _____ | _____ | _____ |
| 19. I felt people didn’t like me. | _____ | _____ | _____ | _____ |
| 20. It was hard to get started doing things | _____ | _____ | _____ | _____ |

Table S5. Comparative analysis of the structural validity of the 20-item CES-DC and the 10-item CES-DC scales.

| **Factors** | **Eigenvalue** | | |
| --- | --- | --- | --- |
|  | Total | % of variance | Cumulative % |
| **20-item CES-DC** | | | |
| 1 | 5.79 | 28.96 | 28.96 |
| 2 | 1.72 | 8.62 | 37.58 |
| 3 | 1.09 | 5.44 | 43.02 |
| 4 | 1.00 | 5.02 | 48.04 |
| 5 | 0.94 | 4.71 | 52.75 |
| 6 | 0.89 | 4.47 | 57.22 |
| 7 | 0.84 | 4.22 | 61.45 |
| 8 | 0.80 | 4.01 | 65.46 |
| 9 | 0.77 | 3.83 | 69.29 |
| 10 | 0.74 | 3.68 | 72.97 |
| 11 | 0.69 | 3.45 | 76.42 |
| 12 | 0.66 | 3.32 | 79.74 |
| 13 | 0.65 | 3.24 | 82.97 |
| 14 | 0.60 | 3.00 | 85.97 |
| 15 | 0.55 | 2.73 | 88.70 |
| 16 | 0.54 | 2.69 | 91.39 |
| 17 | 0.52 | 2.59 | 93.98 |
| 18 | 0.47 | 2.33 | 96.31 |
| 19 | 0.43 | 2.13 | 98.44 |
| 20 | 0.31 | 1.56 | 100.00 |
| **Factors** | **Eigenvalue** | | |
|  | Total | % of variance | Total |
| **10-item CES-DC** | | | |
| 1 | 4.27 | 42.67 | 42.67 |
| 2 | 0.96 | 9.55 | 52.22 |
| 3 | 0.86 | 8.62 | 60.84 |
| 4 | 0.70 | 7.00 | 67.84 |
| 5 | 0.69 | 6.90 | 74.74 |
| 6 | 0.62 | 6.20 | 80.94 |
| 7 | 0.56 | 5.63 | 86.57 |
| 8 | 0.53 | 5.35 | 91.91 |
| 9 | 0.49 | 4.85 | 96.77 |
| 10 | 0.32 | 3.23 | 100.00 |

Table S6. Corrected item-total correlation and Cronbach’s alpha and McDonald´s omega if item deleted.

| **CES-DC 20-item** | Corrected item total correlation | Cronbach’s alpha if item deleted | McDonald´s omega if item is deleted |
| --- | --- | --- | --- |
| **Factor 1. Depressed affect** |  |  |  |
| 14. I felt lonely, like I didn’t have any friends. | 0.633 | 0.800 | 0.799 |
| 19. I felt people didn’t like me. | 0.577 | 0.805 | 0.805 |
| 18. I felt sad. | 0.661 | 0.797 | 0.796 |
| 17. I felt like crying. | 0.642 | 0.797 | 0.797 |
| 6. I felt down and unhappy. | 0.580 | 0.805 | 0.805 |
| 15. I felt like kids I know were not friendly or that they didn’t want to be with me. | 0.467 | 0.817 | 0.818 |
| 3. I wasn’t able to feel happy, even when my family or friends tried to help me feel better. | 0.380 | 0.833 | 0.832 |
| 9. I felt like things I did before didn’t work out right. | 0.545 | 0.808 | 0.808 |
| 10. I felt scared. | 0.398 | 0.827 | 0.826 |
| **Factor 2. Somatic and retardation** |  |  |  |
| 7. I felt like I was too tired to do things. | 0.431 | 0.664 | 0.666 |
| 5. I felt like I couldn’t pay attention to what I was doing. | 0.447 | 0.658 | 0.661 |
| 20. It was hard to get started doing things. | 0.457 | 0.656 | 0.656 |
| 11. I didn’t sleep as well as I usually sleep. | 0.426 | 0.664 | 0.666 |
| 2. I did not feel like eating, I wasn’t very hungry. | 0.352 | 0.682 | 0.683 |
| 1. I was bothered by things that usually don’t bother me. | 0.395 | 0.673 | 0.674 |
| 13. I was more quiet than usual. | 0.373 | 0.679 | 0.682 |
| **Factor 3. Happy** |  |  |  |
| 8. I felt like something good was going to happen. (R) | 0.387 | 0.539 | 0.543 |
| 12. I was happy. (R) | 0.413 | 0.523 | 0.529 |
| 16. I had a good time. (R) | 0.451 | 0.509 | 0.514 |
| 4. I felt like I was just as good as other kids. (R) | 0.346 | 0.589 | 0.593 |
| **CES-DC 10-item** |  |  |  |
| 18. I felt sad. | 0.664 | 0.815 | 0.815 |
| 17. I felt like crying. | 0.657 | 0.814 | 0.813 |
| 14. I felt lonely, like I didn’t have any friends. | 0.609 | 0.820 | 0.819 |
| 19. I felt people didn’t like me. | 0.557 | 0.824 | 0.824 |
| 9. I felt like things I did before didn’t work out right. | 0.566 | 0.823 | 0.823 |
| 6. I felt down and unhappy. | 0.546 | 0.825 | 0.824 |
| 5. I felt like I couldn’t pay attention to what I was doing. | 0.481 | 0.833 | 0.833 |
| 20. It was hard to get started doing things. | 0.456 | 0.835 | 0.835 |
| 1. I was bothered by things that usually don’t bother me. | 0.452 | 0.833 | 0.833 |
| 11. I didn’t sleep as well as I usually sleep. | 0.442 | 0.836 | 0.835 |
| Items are ordered by factor loadings within their factor.  Cronbach's alpha: 20-item CES-DC = 0.852, Factor 1. Depressed affect = 0.828, Factor 2. Somatic and retardation = 0.703, Factor 3. Happy = 0.602; 10-item CES-DC = 0.841  McDonald´s omega: 20-item CES-DC = 0.850, Factor 1. Depressed affect = 0.827, Factor 2. Somatic and retardation = 0.702, Factor 3. Happy = 0.608; 10-item CES-DC = 0.841 | | | |

|  | **1** | **2** | **3** | **4** | **5** | **6** | **7** | **8** | **9** | **10** | **11** | **12** | **13** | **14** | **15** | **16** | **17** | **18** | **19** | **20** |
| --- | --- | --- | --- | --- | --- | --- | --- | --- | --- | --- | --- | --- | --- | --- | --- | --- | --- | --- | --- | --- |
| **1** |  |  |  |  |  |  |  |  |  |  |  |  |  |  |  |  |  |  |  |  |
| **2** | .151** | 1 |  |  |  |  |  |  |  |  |  |  |  |  |  |  |  |  |  |  |
| **3** | .227** | .215** | 1 |  |  |  |  |  |  |  |  |  |  |  |  |  |  |  |  |  |
| **4** | .072 | 0.053 | .042 | 1 |  |  |  |  |  |  |  |  |  |  |  |  |  |  |  |  |
| **5** | .325** | .234** | .243** | .106** | 1 |  |  |  |  |  |  |  |  |  |  |  |  |  |  |  |
| **6** | .287** | .243** | .334** | .109** | .285** | 1 |  |  |  |  |  |  |  |  |  |  |  |  |  |  |
| **7** | .255** | .252** | .172** | .038 | .303** | .267** | 1 |  |  |  |  |  |  |  |  |  |  |  |  |  |
| **8** | 0.061 | .100** | -.015 | .281** | .121** | .111** | .069 | 1 |  |  |  |  |  |  |  |  |  |  |  |  |
| **9** | .383** | .234** | .281** | .181** | .410** | .356** | .303** | .118** | 1 |  |  |  |  |  |  |  |  |  |  |  |
| **10** | .244** | .141** | .152** | .084* | .249** | .275** | .201** | -.012 | .324** | 1 |  |  |  |  |  |  |  |  |  |  |
| **11** | .245** | .201** | .204** | .037 | .258** | .281** | .290** | .082* | .245** | .225** | 1 |  |  |  |  |  |  |  |  |  |
| **12** | .138** | .066 | .070 | .241** | .119** | .186** | .099* | .262** | .227** | 0.062 | 0.068 | 1 |  |  |  |  |  |  |  |  |
| **13** | .207** | .240** | .223** | .009 | .228** | .299** | .190** | -.002 | .196** | .181** | .256** | .015 | 1 |  |  |  |  |  |  |  |
| **14** | .309** | .263** | .278** | .159** | .301** | .469** | .243** | .080* | .427** | .257** | .310** | .170** | .262** | 1 |  |  |  |  |  |  |
| **15** | .270** | .166** | .249** | .156** | .220** | .360** | .181** | .090* | .307** | .227** | .226** | .146** | .240** | .420** | 1 |  |  |  |  |  |
| **16** | .126** | .123** | .152** | .247** | .153** | .278** | .150** | .311** | .235** | .127** | .105** | .437** | .097* | .271** | .253** | 1 |  |  |  |  |
| **17** | .308** | .242** | .250** | .092* | .358** | .427** | .291** | .056 | .388** | .399** | .377** | .191** | .278** | .510** | .283** | .237** | 1 |  |  |  |
| **18** | .282** | .278** | .295** | .119** | .328** | .440** | .272** | .095* | .432** | .341** | .378** | .220** | .270** | .505** | .294** | .308** | .668** | 1 |  |  |
| **19** | .271** | .230** | .250** | .201** | .284** | .408** | .215** | .068 | .390** | .200** | .234** | .194** | .202** | .482** | .391** | .254** | .474** | .491** | 1 | . |
| **20** | .269** | .232** | .197** | .084* | .296** | .293** | .294** | .043 | .302** | .357** | .310** | 0.059 | .261** | .280** | .297** | .201** | .333** | .341** | .283** | 1 |
| ^*^*p* < .05. ^**^*p* < .01 | | | | | | | | | | | | | | | | | | | | |

Table S7. CES-DC scale inter-item correlation.

|  | 20-item CES-DC | | 10-item CES-DC | |
| --- | --- | --- | --- | --- |
|  | β ± SD | p-value | β ± SD | p-value |
| BMI | 0.24 ± 2.17 | **0.004** | 0.16 ± 1.30 | **0.002** |
| Waist circumference | 0.07 ± 0.75 | **0.015** | 0.04 ± 0.45 | **0.017** |
| CRF | -0.19 ± 1.79 | **0.006** | -0.10 ± 1.09 | **0.017** |
| HRQoL | -0.51 ± 0.66 | **< 0.001** | -0.27 ± 0.43 | **< 0.001** |
| Physical wellbeing | -0.23 ± 0.84 | **< 0.001** | -0.10 ± 0.52 | **< 0.001** |
| Psychological wellbeing | -0.54 ± 0.65 | **< 0.001** | -0.29 ± 0.41 | **< 0.001** |
| Autonomy and parent relation | -0.38 ± 0.72 | **< 0.001** | -0.19 ± 0.46 | **< 0.001** |
| Peer relations | -0.33 ± 0.83 | **< 0.001** | -0.17 ± 0.51 | **< 0.001** |
| School environment | -0.37 ± 0.72 | **< 0.001** | -0.20 ± 0.45 | **< 0.001** |
| Abbreviations: BMI = body mass index; CRF = cardiorespiratory index; HRQoL = health related quality of life. N = 671.  Data are presented as beta coefficient (β) ± standard deviation (SD). The values in bold indicate statistical significance at p < 0.05. | | | | |

Table S8. Linear regression models among 20-item CES-DC and 10-item CES-DC and measures of anthropometry, fitness, and quality of life.

Table S9. Concordance between 20-item CES-DC and 10-item CES-DC for the screening of depression in children with cut-off for the 10-item CES-DC set at 7.

| 20-item CES-DC^a^ | 10-item CES-DC^b^ | | Total |
| --- | --- | --- | --- |
|  | Non-depressed | Depressed |  |
| Non-depressed | 402 (59.9%) | 47 (7.0%) | 449 (66.9%) |
| Depressed | 21 (3.1%) | 201 (30.0%) | 222 (33.1%) |
| Total | 423 (63.0%) | 248 (37.0%) | 671 (100%) |

Note. Data are presented as n and %. Cohen´s Kappa coefficient = 0.78.

aCut-off for the 20-item CES-DC-20 was set at 15.

bCut-off for the 10-item CES-DC was set at 7.

Figure S1. Diagram flow of the study participants in the current study.

Invited for participation:

1,049

Non-respondents:

304 actively refused or did not return the written approval.

Respondents:

745

Excluded:

74 had missing data on 20-item CES-DC scale

Population for analyses:

671

Figure S2. Sedimentation graph of factor components of 20-item CES-DC and 10 item CES-DC.

### **A.**


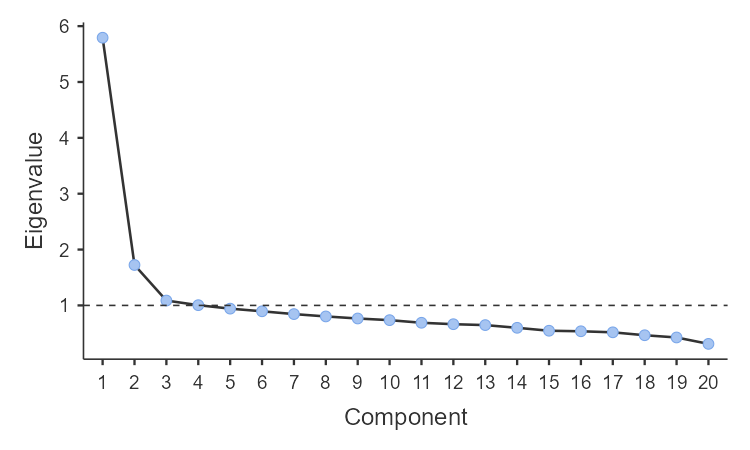


### **B.**


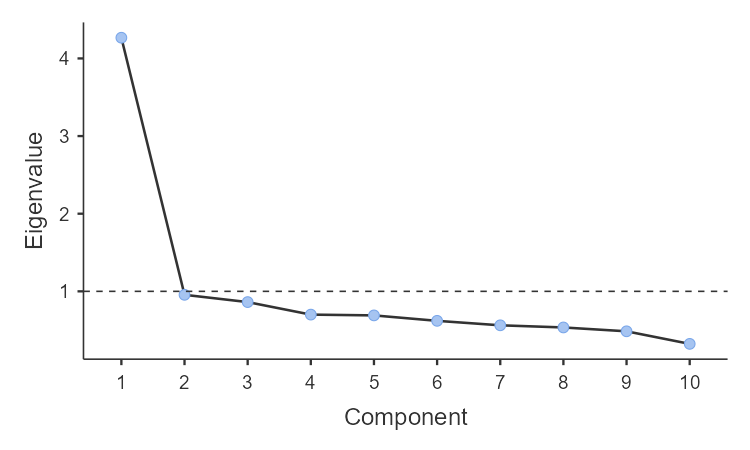


*Note.* A: sedimentation graph of 20-item CES-DC. B: sedimentation graph of 10-item CES-DC.

**Figure S3. Parallel analysis of 20-item CES-DC and 10-item CES-DC.**

**A.**


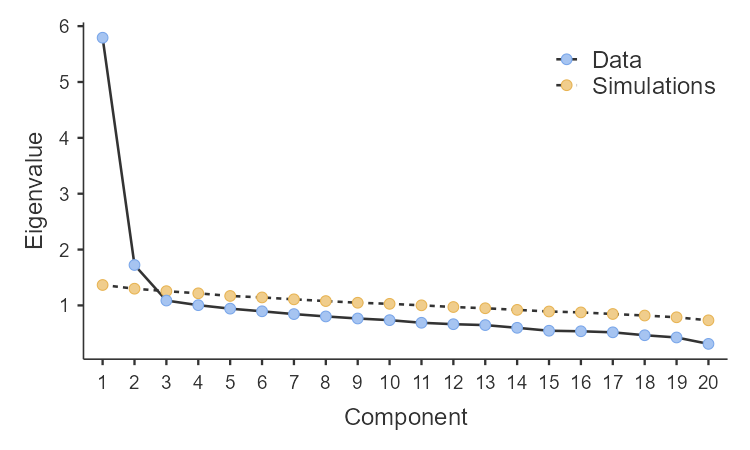


**B.**


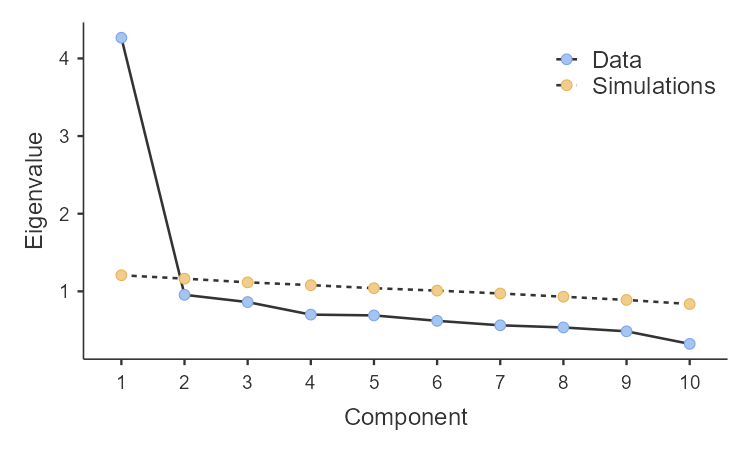


*Note.* A: 20-item CES-DC. B: 10-item CES-DC.


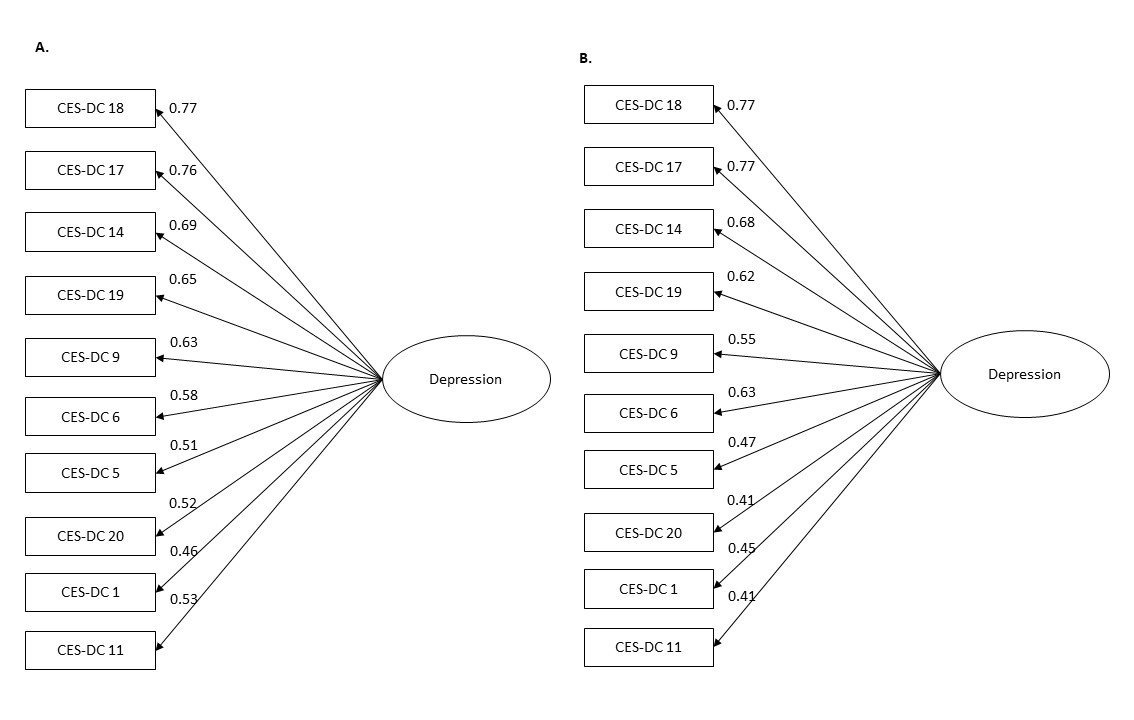
 Figure S4. Factor loading and goodness of fit of CES-DC 10 by sex.

*Note.* Figure A = boys’ sample, n = 332; Figure B = girls’ sample, n = 339. χ2 = 198.97; df = 70; p < 0.001; CFI = 0.94 and RMSEA = 0.052.

Figure S5. Factor loading and goodness of fit of CES-DC 10 by body mass index cut-offs.


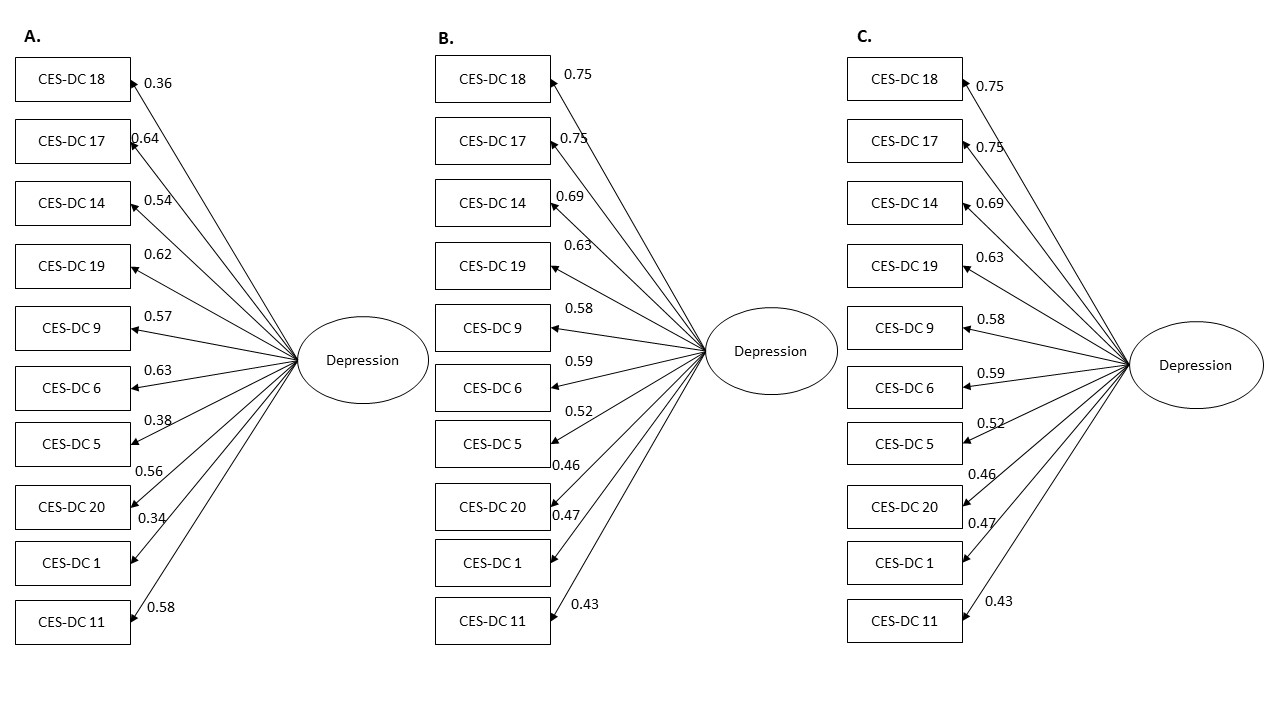


*Note.* Figure A: low weight, n = 59; Figure B: normal weight, n = 406; Figure C: overweight/ obesity, n = 206. χ^2^ = 257.93; df = 105; p < 0.001; CFI = 0.92 and RMSEA = 0.047.


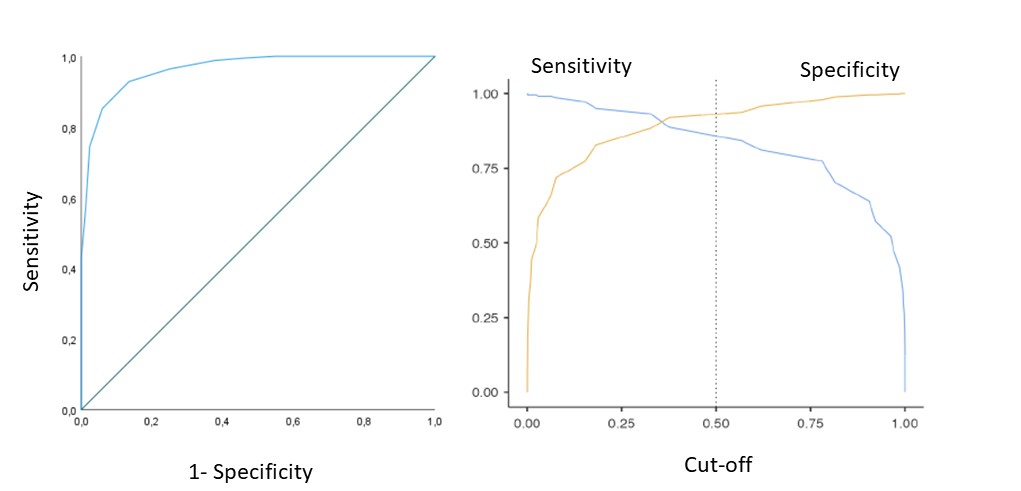
Figure S6. Receiver Operating Characteristics (ROC) curve and cut-off plot for the 10-item CES-DC in comparison with 20-item CES-DC.

*Note*: AUC = 0.97.
